# Computer Aided Identification of Small Molecules Disrupting uPAR/α5β1- Integrin Interaction: A New Paradigm for Metastasis Prevention

**DOI:** 10.1371/journal.pone.0004617

**Published:** 2009-02-26

**Authors:** Pratima Chaurasia, Mihaly Mezei, Ming-Ming Zhou, Liliana Ossowski

**Affiliations:** 1 Division of Hematology/Oncology, Department of Medicine, Mount Sinai School of Medicine, New York, New York, United States of America; 2 Department of Structural and Chemical Biology, Mount Sinai School of Medicine, New York, New York, United States of America; Ordway Research Institute, United States of America

## Abstract

**Background:**

Disseminated dormant cancer cells can resume growth and eventually form overt metastases, but the underlying molecular mechanism responsible for this change remains obscure. We previously established that cell surface interaction between urokinase receptor (uPAR) and α5β1-integrin initiates a sequel of events, involving MAPK-ERK activation that culminates in progressive cancer growth. We also identified the site on uPAR that binds α5β1-integrin. Disruption of uPAR/integrin interaction blocks ERK activation and forces cancer cells into dormancy.

**Methods and Principle Findings:**

Using a target structure guided computation docking we identified 68 compounds from a diversity library of 13,000 small molecules that were predicted to interact with a previously identified integrin-binding site on uPAR. Of these 68 chemical hits, ten inhibited ERK activation in a cellular assay and of those, 2 compounds, *2-(Pyridin-2-ylamino)-quinolin-8-ol and*, *2,2′-(methylimino)di (8-quinolinol)* inhibited ERK activation by disrupting the uPAR/integrins interaction. These two compounds, when applied *in vivo*, inhibited ERK activity and tumor growth and blocked metastases of a model head and neck carcinoma.

**Conclusions/Significance:**

We showed that interaction between two large proteins (uPAR and α5β1-integrin) can be disrupted by a small molecule leading to profound downstream effects. Because this interaction occurs in cells with high uPAR expression, a property almost exclusive to cancer cells, we expect a new therapy based on these lead compounds to be cancer cell specific and minimally toxic. This treatment, rather than killing disseminated metastatic cells, should induce a protracted state of dormancy and prevent overt metastases.

## Introduction

There is long standing clinical evidence, some inferential, but recently at the molecular level, that disseminated cancer cells can persist in a patient in a dormant state with no symptoms before they become actively growing to form overt metastases [1]. However, the mechanisms responsible for the conversion from dormant to active state remain largely unknown. Our long term goal is to identify molecular events that control cancer dormancy and to find ways to mimic them with aim of inducing and maintaining dormancy.

Previously, using a model of head and neck cancer, we determined that a high ratio of MAPK-ERK to SAPK-p38 activity was necessary for these cells to form a progressively growing tumor [2]. This ratio was subsequently found to be predictive of the ability to form tumors by cells from multiple cancers [1]. In the head and neck model this ratio was achieved through the interaction of α5β1-integrin with urokinase receptor (uPAR) which is highly expressed on these cells [3]. We showed that this interaction activated the integrins [2] and that through an “outside in” activation process, EGFR was recruited to the complex and activated signaling to ERK [4]. We also showed that antibody directed to Domain III region of uPAR, a protein made of 3 folded domains, but not other anti-uPAR antibodies, disrupted the uPAR/integrin interaction.

Before initiating a search for small molecules capable of disrupting uPAR/integrins association, we first identified the site on uPAR to which the integrin bound; this site is located at residues 240–248 of Domain III of uPAR [5]. The solved crystal structure shows that the 3 folded domains of uPAR form a urokinase binding pocket in the front of the molecule [6] while residues 240–248 are located in the back of the structure. Treatment of tumorigenic cells that possess a functional uPAR-integrins complex with a peptide derived from uPAR residues 240–248 disrupted the complex, inactivated the α5β1-integrin, and reduced signal to ERK, while the same peptide with S245A substitution was inactive. The fact that a single amino acid was responsible for the functionality of the complex increased the possibility that a small molecule will be able to disrupt the interaction.

Although, an antibody and a peptide are able to disrupt the interaction, we believe that a small molecule with the same activity would be preferable. Since disruption of uPAR/integrin interaction shifts cells into proliferative arrest, without causing their death, to be an effective treatment the drug would have to be used chronically in patients suspected to have overt disseminated or residual disease. For that reason it would have to have limited toxicity and, 0preferably, be orally available. Because the library was selected on the basis of Lipinski's rule of 5 [7], and because of the target which is an interaction that takes place when uPAR is over-expressed, a condition mostly of malignant tumors, those two requirement should be satisfied.

Our experimental model assigns an important role to a highly expressed uPAR in regulating cancer dormancy [2], [3], [8]. It fits well with the observations that high uPAR expression predicts for more aggressive disease in several cancer types [9]–[14] and that circulating and bone marrow cancer cells express uPAR [15]–[17]. Simultaneous uPAR and HER2/neu gene amplification on circulating cancer cells has also been described [14]. In gastric cancer uPAR expression on cancer cells in bone marrow is a prospective predictor of proliferation of these cells and shorter patient survival [18]. These considerations prompted our search for small molecules that might target the uPAR/integrin interaction. We conducted computational screening of a diversity library of ∼13,000 small molecules using the uPAR crystal structure [6] with a focus on the residues 240–248 region, which we showed to be important for integrin interaction. This screen identified 68 compounds, which were further characterized for their effect on ERK inhibition using a tester cell line in which luciferase expression was under the control of activated ERK.

Here we report evidence that *2-(Pyridin-2-ylamino)-quinolin-8-ol* and its analog, *2,2′-(methylimino)di (8-quinolinol)* were able to functionally disrupt uPAR/integrin interaction. As a consequence of the uPAR/integrin disruption, reflected in reduced ERK activity, a significant inhibition of tumor growth was observed with almost complete inhibition of metastases.

## Results

### *In silico* screening of a diversity library of small molecules on uPAR

The *in silico* screening of a library of chemical compounds for their potential to disrupt uPAR/integrins interaction is the culmination of a long effort to find ways to induce cancer cell dormancy. It was made possible by our identification of an integrin binding sequence on uPAR [5] and the recently published crystal structure of uPAR [6].

**Figure 1:**
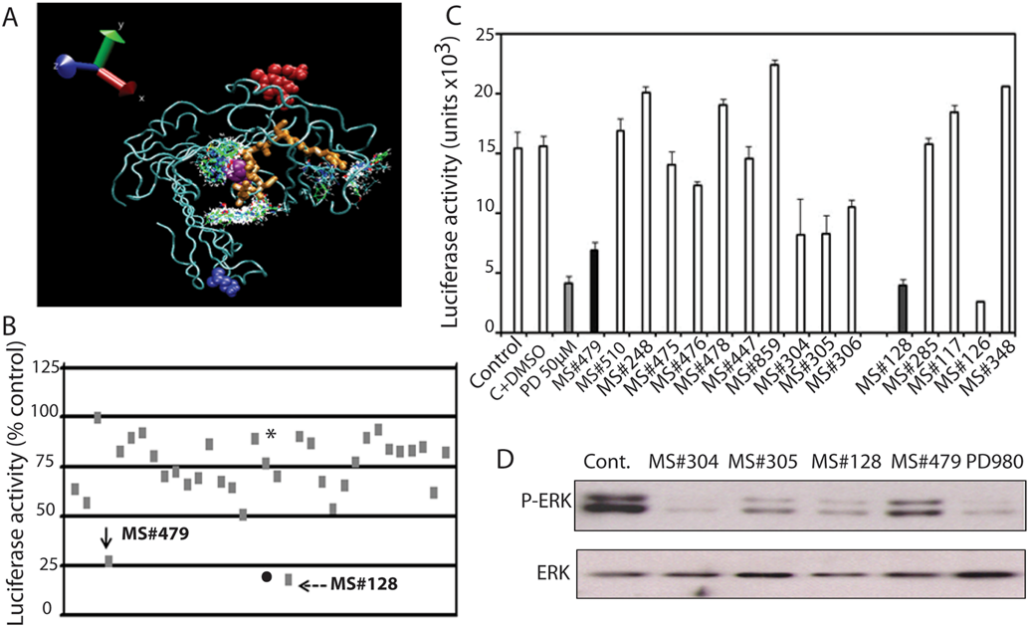
*In silico* docking of small molecule library and analysis of top compounds. A. Docking of small molecule library onto uPAR. In-silico screen of a diversity library selected on the basis of the Lipinski rule using Autodock (v 3.0.5) for possible binders to uPAR, targeting the region comprising residues 240–248. B. Test of top-scoring compounds for ERK inhibition. T-HEp3 cells stably transfected with a plasmid expressing Elk1-GAL4 fusion protein and plasmid expressing GAL4UAS-TATA-luciferase (pD700-luciferase), plated in 96 wells plates were treated overnight with 5 µM of the top-scoring compounds generated by *in silico* docking of library of compounds on uPAR^240–248^ sequence. The cells were lysed and Luciferase activity was measured in triplicates. The numbers (mean of 3 determinations) show luciferase as % of diluents (DMSO) treated control. *- PD98059, 5 µM, •−50 µM. C. Structure activity relationship analysis of compounds MS#479 and MS#128. Promising ligands (MS#479 and MS#128) were entered into the ZINC database of over 4.6×10^6^ small molecules, and commercially available analogs were selected for further testing. The testing was as described for 1B. Each bar is the mean of 3 determinations. The experiment was repeated twice. D. Compounds that inhibit luciferase activity also inhibit P-ERK. T-HEp3 cells transfected as in B, were treated with 20 µM of compound MS#479, 305, 304 and 128 for 20 min, lysed and tested for P-ERK by Western blotting. PD98059 (10 µM) served as a positive control.

A diversity library of about 13,000 small molecules was screened using Autodock (v 3.05) for possible binders to uPAR and to the specific site on uPAR that binds integrin α5β1. The input describing the protein was prepared with the program Autodock Tools (ADT); it involved adding charges and non-bonded parameters to the protein structure file and orienting the protein to minimize the enclosing rectangle using an in-house program, Simulaid. The screening and the filtering of the docked poses were driven, respectively, by a script and a program (Dockres). Of the top-scoring molecules that docked on uPAR (68 in total) 32 showed preferential docking on the sequence consisting of residues 240–248 (Fig. 1A) and those were further tested in a cell-based assay.

We used a head and neck cancer (HNSCC) cell line, T-HEp3, which expresses high level of uPAR and α5β1-integrin, which by interacting are responsible for generating *in vivo* ERK activation and proliferative signal [2]. We stably transfected the cells with 2 plasmids; pFA (*Elk_AD_-GAL4_DBD_*), which encodes for a fusion protein that transactivates, when phosphorylated by ERK, a second plasmid, pD700 (5X-GAL4UAS-tk-luciferase). These cells were incubated for 16 hrs with 5 µM of the selected compounds, lysed and tested for Luciferase activity. Luciferase activity inhibition as percent of untreated control (Fig. 1B), and thus ERK inhibition, by two of the compounds, MS#479 and MS#128 was found to be similar to the inhibition of ERK by a MEK inhibitor, PD98059, but the former two compounds were at least 10 fold more effective than PD98059 (Fig. 1B). (At 5 µM PD98059 was ineffective, Fig. 1B). From a search of the ZINC database that contains over 4.6 million small molecules, we found 10 and 4 commercially available analogs for MS#479 and MS#128, respectively, which were tested using the same Luciferase activity assay as in Fig. 1B. Compared to the control, the 3 analogs of the MS#479 compound inhibited Luciferase by at least 30%, but less than the original compound which inhibited by at least 60%, while one of the MS#128 analogs, MS#126, inhibited Luciferase, and thus ERK-activity by 82% and slightly more than the original compound (75%) (Fig. 1C).

To confirm that the observed drop in Luciferase activity was indeed due to a reduction in ERK activity, we treated T-HEp3 monolayer cells with 20 µM of several of the active compounds for 45 min, lysed the cells and determined P-ERK and ERK content by Western blotting. As shown in Fig. 1D, short incubation with the compounds led to a profound inhibition of P-ERK content, in some cases similar to the effect of an established MEK inhibitor, PD98059.

Treatment of 2 melanoma cell lines, UCT-2 and A2058, both of which express α5β1-integrin and have highly activated ERK induced by mutated B-RAF, but no uPAR expression (Estrada, Y. Dong, J-L. and Ossowski, L., Pigment Cell Melanoma Research, In Press), with 20 µM of MS#479 for 1 hr, did not cause reduction in P-ERK by Western blot analysis while PD treatment inhibited P-ERK (Results not shown), further confirming the specificity of the uPAR/integrin as a target.

### Mechanism of ERK inhibition by compounds

**Figure 2:**
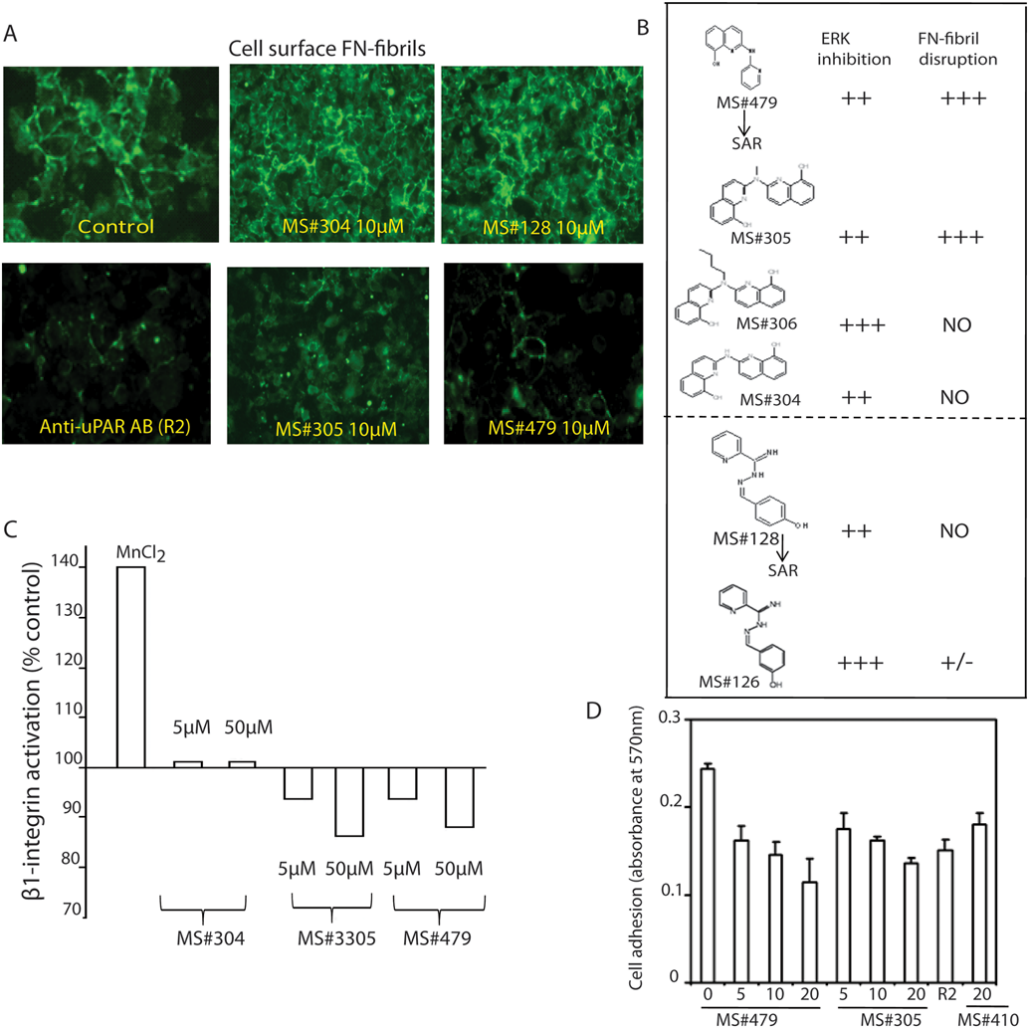
Testing ERK-inhibiting compounds for their effect on uPAR/integrin interaction. A. Inhibition of FN-fibril formation. T-HEp3 cells in suspension were incubated with compounds MS#479, MS#305, MS#304 or MS#128 (10 µM) in DMEM with 5% FN-free FBS for 15 mins, inoculated into chambered slides and incubated overnight with DMEM with 10 µg/ml human FN. The cell-bound FN fibrils were detected by IF as described in Methods. This experiment was repeated 3 times. B. Structure of compounds tested for Luciferase and FN-fibril inhibition. Luciferase was examined as in Fig. 1B and fibril disruption as in Fig. 2A. C. Inactivation of β1-integrin. Monolayers of T-Hep3 cells were incubated with 1 mM MnCl_2_, or 5 or 50 µM of compound MS#304, MS#305, or MS#479, cells were detached and incubated with 5 µg/ml antibody to active β1-subunit of integrin (HUTS-4), or IgG2b, followed by anti-mouse IgG coupled with Alexa 488 and analyzed by FACS. The bars show mean fluorescence intensity as percent of untreated control. This experiment was repeated twice with similar results. D. Inhibition of adhesion to FN. T-HEp3 cells were treated in suspension with anti-uPAR domain III (R2) antibody (15 µg/ml), or with 5, 10 and 20 µM of compound MS#479 or MS#305 or with 20 µM compound MS#410 as negative control and adhesion was determined after 15 min at 37°C as previously described [5]. The results are mean (SD) of 4 determinations. The results are statistically significant (ANOVA p<0.0001). This experiment was repeated twice with similar results.

As stated earlier, uPAR/integrin interaction leads to ERK activation, a process responsible for *in vivo* proliferation of T-HEp3 cells [2], [3]. Disruption of this interaction, a tumor cell specific target, causes α5β1-integrin inactivation and forces cancer cells into a state of dormancy [2], [8]. One important indication of α5β1-integrin activation is its ability to bind fibronectin (FN) and to organize it into insoluble fibrils on the cell surface [2], [5], [8]. To identify among ERK inhibitors those that function through inactivation of α5β-integrin, we tested the effect of the “docked” compounds (Fig. 1A) that inhibited ERK (Fig. 1C), as well as their non-inhibitory analogs (Fig.S1), for their ability to interfere with FN-fibril formation. T-HEp3 cell bound insoluble FN-fibrils were detected by immunofluorescence (IF). After 16 hrs of incubation at 37°C we found that approximately 60% of cells in control cultures had fibrils on their surface (Fig. 2A). The anti-uPAR antibody, R2, which we previously showed to block fibril formation [8], was again inhibitory. Of the 6 ERK inhibiting compounds tested, only 2, MS#479 and MS#305 showed a dose-dependent ability to block fibrils, and at 10 µM were nearly as efficient as the anti-uPAR antibody. The rest of the compounds, including 4 additional ERK inhibitors, (Fig. 2B) did not block fibril formation (Fig. 2A and 2B), suggesting that they inhibit ERK through a different mechanism. The two compounds, MS#479 and MS#305 had a slight growth inhibitory effect on T-HEp3 cell in culture when tested at 10 µM for longer than 40 hrs and at 7.5 µM for longer than 72 hrs.

To test directly the effect of compounds MS#479 and MS#305 on α5β1-integrin inactivation, T-HEp3 cells were incubated with or without 1 mM MnCl_2_, or with 5 and 50 µM of the two active compounds; MS#304, which inhibits ERK but does not disrupt fibrils, served as negative control. Cells were then incubated with HUTS-4 antibody that recognizes the active conformation of β1 integrin [19], or with an isotype matched IgG, followed by secondary rabbit-anti mouse IgG antibody coupled to Alexa 488 and examined by FACS. As expected, (Fig. 2C), MnCl_2_ treatment stimulated HUTS-4 binding by ∼40% over control, while the 2 lead compounds inhibited HUTS-4 binding by ∼15%; MS#304 had no effect. Several other ERK inhibiting compounds that did not cause FN-fibril disruption were found to be ineffective in integrin inactivation as determined by HUTS-4 antibody binding (Results not shown).

We next tested whether the two compounds, by inactivating the integrin will interfere with cell adhesion and spreading. Reduction of adhesion might be important in depriving cells *in vivo* of a pro-proliferative/survival signal and in reducing their ability to migrate. Suspensions of T-HEp3 cells (1.5×10^4^) were treated for 10 min with increasing concentrations of the two lead compounds and with an inactive compound MS#410 and inoculated into FN-coated wells. (We used MS#410 because it does not disrupt uPAR/integrin and does not inhibit ERK). Cells treated with anti-uPAR antibody served as a positive control. Cells that attached and spread after 15 min of incubation at 37°C were quantified. The two lead compounds reduced cell adhesion to FN significantly (p<0.01), and in a dose dependent fashion, while the inactive compound tested at the highest concentration (20 µM) had no effect (Fig. 2D).

### Disruption of physical interaction of uPAR/integrin complex by MS#479

**Figure 3:**
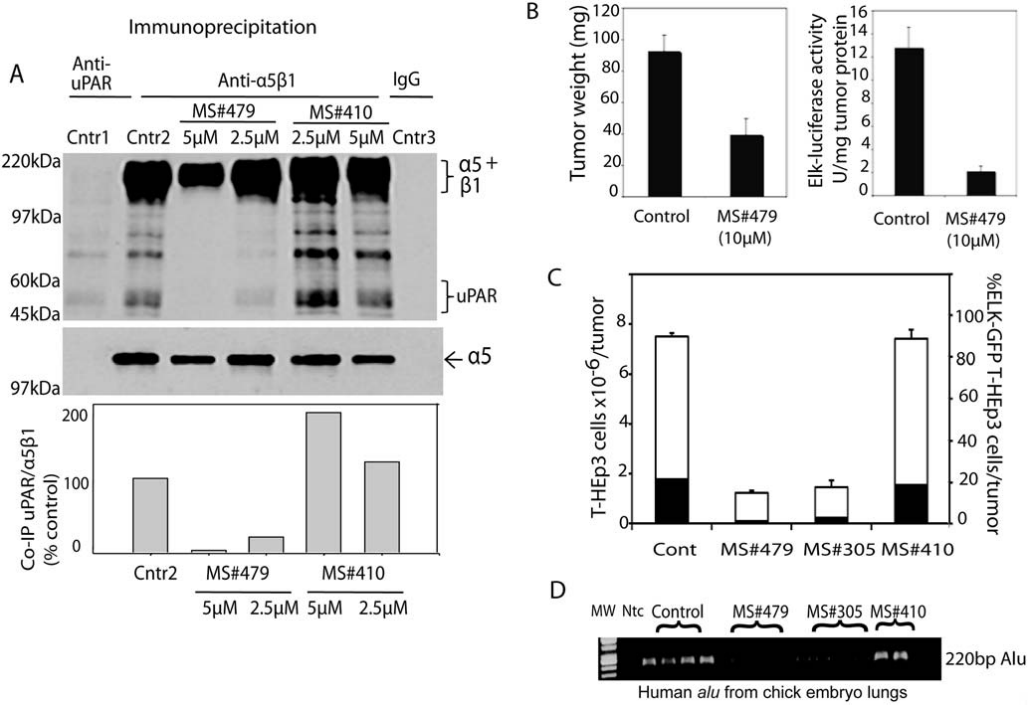
Lead compounds inhibit tumor growth and metastasis through uPAR/integrin disruption and ERK inhibition. A. Disruption of physical interaction between uPAR and α5β1-integrin. Confluent T-HEp3 cells were surface biotinylated, lysed and incubated with Domain I anti-uPAR antibodies R3 (Cntr1), anti-α5β1-integrin antibody (HA5), or with isotype matched IgG, (Cntr 3). After 1 hr the precipitates were treated for 15 min with 2.5 or 5.0 µM of compound MS#479 or MS#410 and the proteins analyzed by Western blotting using Neutravidin-HRP conjugate for detection. Bottom panel; top portion of the upper blot was re-probed, after stripping, with anti-α5 integrin antibody. α5-integrin and uPAR were scanned and quantified using Image J and the ratio of uPAR to integrin was determined. The bars represent the ratio expressed as percent of control. This experiment was performed 3 times. B. In vivo treatment of T-HEp3 cells with compound MS#479 inhibits ERK activity and tumor growth. T-HEp3 cells stably transfected with ELK-luciferase (as in Fig. 1C) were mixed with 10 µM of compound MS#479 in DMSO or DMSO (0.1%) and inoculated on CAMs of 10 day old chick embryos at 4×10^5^ cells/CAM, 4 CAMs per group. The tumors were treated daily with 30 µl of 10 µM MS#479 or 30 µl of 0.1% DMSO for 6 days, at which time the tumors were excised, weighed, lysed and Luciferase activity was determined as described in Methods. The inhibition by compound #479 was statistically significant (n = 7/group, tumor weight, p<0.0001, Luciferase, p<0.0001, t-test). C. The effect of MS#479, MS#305 and MS#410 (negative control) on tumor growth and ERK inhibition measured by ELK-GFP expression. T-HEP3 cells stably transfected with ELK-GFP construct were treated with the 3 compounds or DMSO as in B but 5×10^5^ were inoculated and, at the end of the incubation, single cell suspensions of tumors were prepared and total as well as GFP-expressing cell number was determined for each tumor. The graph shows the total number of tumor cells per tumor (mean, n = 5, white bars) and percent of GFP-positive cells (darkened part of bars). Both compounds significantly (ANOVA p<0.0001) inhibit tumor growth. MS#410 has no effect. D. The effect of compounds on spontaneous metastasis. To quantify metastases, lungs were removed from chick embryos in experiment described in Fig. 3C, DNA was extracted and used to amplify a 220 bp fragment of human *alu* sequence as previously described [21]. One of the MS#410 treated embryos was dead and 1 showed toxic effects.

To test the ability of compounds to physically disrupt the uPAR/integrin complex, T-HEp3 cells were surface-biotinylated, lysed and the complex immunoprecipiated by anti-uPAR domain I antibody (R3) or by anti-α5β1 integrin antibody in the presence of 0, 2.5 or 5 µM of compound MS#479, or an inactive compound MS#410. Because the R3 antibody does not efficiently immunoprecipitates uPAR, to show uPAR association with α5β1-integrin using R3 required very long film exposures (Results not shown). The proteins, resolved on PAGE, were probed with Neutravidin after transfer; the integrin bands were also probed with anti-α5 antibody. The results (Fig. 3A) represented as uPAR/integrin ratio are expressed as percent of untreated control. Treatment with 2.5 µM of compound MS#479 reduced the uPAR/integrin association by more than 70%, and 5 µM blocked it altogether. This effect was more potent than the previously shown disruption of uPAR/integrin association by 50 µM of uPAR-derived peptide residue 240–248 [5]. (Peptide 240–248 with alanine in place of serine 245 was completely inactive [5]). An inactive MS#479 analog (compound MS#410) did not disrupt the complex. Another active compound, MS#305 produced similar results to MS#479, but at 5 and 10 µM, respectively (Results not shown).

### Compounds MS#479 and MS#305 inhibit tumor growth

We previously showed that uPAR with serine 245 to alanine mutation was neither able to interact with the integrin nor to stimulate *in vivo* proliferations [5]. We tested whether compounds that docked to this sequence and showed potent *in vitro* disrupting activity will affect the *in vivo* growth. T-HEp3 cells stably transfected with plasmids Elk_AD_-GAL4_DBD_ and the 5X-GAL4UAS-luciferase were pre-incubated for 20 min with 10 µM MS#479, or the diluents alone, and inoculated on the chorioallantoic membranes (CAMs) at 4×10^5^ cells/CAM, treated daily with the compound for 5 consecutive days, excised, weighed, lysed and the lysates used for Luciferase measurements. As shown in Fig. 3B, left panel, MS#479 treatment inhibited tumor weight by ∼60% (p = 0.0001, t-test), and Luciferase activity, expressed as units per mg of tumor protein, by ∼85% (p = 0.003 t-test)(Fig. 3B, right panel). Because Luciferase expression in this cells is under control of uPAR/integrin activated ERK, these results show that MS#479, by inhibiting ERK, inhibits tumor growth.

### Can MS#479 and MS#305 prevent spontaneous metastasis?

To study the effect of uPAR/integrin disrupting compounds on spontaneous metastasis we used stably transfected T-HEp3 cells, similar to the one described above but in which the 5X-GAL4UAS promoter was linked to hrGFP. We have shown previously that T-HEp3 cells disseminate rapidly and predictably to multiple organs when inoculated on the CAM [20] and that PCR amplification of human *alu* sequences in DNA extracted from chick embryo organs, including lungs, is an accurate measure of the number of disseminated cancer cells [21]. We thus mixed the T-HEp3 cells with 10 µM of MS#479, MS#305 and, as negative control, MS#410, inoculated the cells into Teflon rings placed on CAMs and continued the daily treatments for additional 5 days. This treatment did not interfere with cell attachment to the CAM, possibly because the CAM, in addition to FN, the ligand for α5β1-integrin contains multiple other extracellular membrane proteins, such as collagen and vitronectin that might mediated HEp3 cell attachment. The percent of GFP positive cells was determined in single cell suspensions of tumors using an inverted fluorescent microscope. Fig. 3C, shows that both MS#479 and MS#305produced strong (>60%) and significant (p<0.0001, ANOVA) inhibition of tumor growth. In both cases, ELK-GFP tracer cells, in which the GFP is proportional to the level of ERK activation, reliably tracked the effect on overall tumor growth (Fig. 3C, blackened part of the bars).

To test for the effect of compounds on metastases, DNA was extracted from chick embryo lungs and their content of disseminated cancer cells was measured by human *alu*-PCR amplification as described previously [21]. Fig. 3D shows that treatment with MS#479, results in a potent reduction in lung-human *alu* sequences, indicating the presence of fewer disseminated cancer cells. MS#305 has somewhat lesser effect and the inactive MS#410 is not inhibitory. This experimental approach does not permit to conclude whether the effect on metastasis is due to reduction in primary tumor size, an effect on dissemination due to reduced migration or inhibition of cancer cells proliferation in the secondary sites.

**Figure 4:**
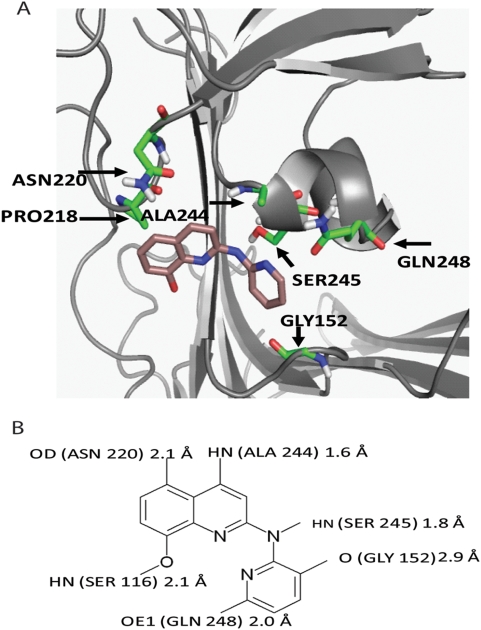
Chemical structure of the lead compound and its interaction in the binding site in uPAR. A. Top-scoring pose of the ligand MS#479 (magenta) shown in its binding pocket in uPAR. Side chains of polar residues within 3Å of the ligand are highlighted; the rest of the protein is displayed in a ribbon diagram. B. Hydrogen-bonding interactions. Polar atoms of the protein shown within 3Å of the ligand suggest corresponding derivatization for improvement of ligand binding affinity.

We have previously shown that in the case of uPAR/integrin interaction, serine 245 was crucial for this interaction [5]. Indeed we found that serine 245 of a small α-helix provides a crucial site of molecular interaction (see Fig. 4A). It is located in a surface exposed cavity that is lined with amino acid residues carrying various functional groups capable of hydrogen bonding, electrostatic and hydrophobic interactions. Of these Pro 218, Asn 220, Ser 245, Gln 248, Ala 244 interact with the lead compound. Polar atoms of the protein located within 3Å of the ligand (Fig. 4B) suggest possible derivatives for improvement of ligand affinity. Once such derivatives of medicinal quality are available, we will examine the mechanism of dormancy induction in disseminated cells.

## Discussion

The main goal of this study was to identify small molecules that by disrupting interaction between two receptors and inverting the ratio between active ERK and p38 will force disseminated cancer cells into a state of dormancy. This was based on our previous work that delineated a positive loop leading from uPAR/integrin interaction through ERK activation to transcriptional up-regulation of uPAR and determined that interruption of this loop forces cancer cells into dormancy [2]–[5], [8]. We now identified two small molecules with uPAR/integrin disrupting activity that reduce ERK activation and, when applied to cancer cells *in vivo*, inhibit growth.

Why is this approach preferable to direct blocking of ERK activity by MEK inhibitors, or to blocking of integrin activation by antagonists? The first and most important advantage is the relative specificity of the target; an interaction that takes place in high uPAR-expressing cells, such as cancer cells. The intended use of these compounds is to prevent occurrence of overt metastases in patients that harbor clinically undetectable residual disease. Since the drug is expected to induce and maintain dormancy, rather than cause acute cancer cell death, a successful outcome implies prolonged treatments and thus the need for oral availability and high index of specificity for cancer cells. We pose that inhibiting ERK by disrupting uPAR/integrin interaction should be less toxic than direct blocking of ERK activity through MEK inhibitors or inactivation of integrins, both of which would have more broader targets.

We and others have repeatedly shown that uPAR directly interacts with integrins affecting their activation state and changing intracellular signaling [3], [14], [22]–[26]. This has been shown by FRET, co-immunoprecipitation (IP) and genetic modifications of uPAR or the integrin. Only in specific instances involving vitronectin as matrix, the role of uPAR on integrin function was shown to be indirect [27]. Since uPAR does not directly bind to FN, the preponderance of published data, including our own, allows the conclusion that MS#479 disrupts the existing interface between uPAR and the integrin and stops the signaling cascade. This has been confirmed by multiple approaches, including FN-fibril disruption, reduced adhesion to FN, and loss of “activation” epitopes on integrins (Fig. 2), which all point to “de-activation” of the integrin. Cells that express α5β1-integrin but not uPAR, such as melanoma, were insensitive to the effect of MS#479. We did not examine the effect of the two lead compounds on disruption of other uPAR/integrin pairs. However, our previous work has shown [5] that α3β1-integrin binds much less efficiently to the identified 240–248 amino acid residue sequence. Because other uPAR/integrin complexes might also be important in generating proliferative signals in cancer cells, it might be necessary to find specific molecules that will disrupt these interactions.

Is it feasible that a 3-domain folded uPAR protein can physically contact α5β1-integrin through a sequence in domain III, which most likely is membrane proximal? In spite of extensive effort by several outstanding laboratories, some controversies regarding integrin conformation capable of ligand binding and the mechanism of their activation still persist [28]. Although, it is believed that integrin is in extended form when binding ligands, there is also evidence based on electron microscope analysis indicating that the extodomain of integrin bound to a fragment of fibronectin (F7–F10) appears to be in a similar compact (bent) conformation as the unbound extodomain [29]. This suggests that ligands such as uPAR, smaller than the full length large matrix proteins, can bind to the bent conformation. Whether this is sufficient to fully activate integrin or whether additional interactions need to take place, remains to be determined.

In our search for active compounds we combined biological and biochemical approaches to identify the site of interaction between the two receptors. We found the sequence 240–248, (GCATASMCQ) to be the site of interaction, with serine 245 being a crucial residue. This information facilitated the *in silico* screening of a library of compounds in that the computational evaluation of chemical compound binding to the target protein could be analyzed in greater detail. To the best of our knowledge the disruption by a small molecule of extracellular domains of two cell surface receptors and its consequence on intracellular signaling and biological outcomes has not been described, adding to the novelty of our approach. It was previously thought that due to the large surfaces area involved in protein/protein interface [30], small molecules would not be optimal candidates for disruption of these interactions. However, more recently, it was concluded that a much smaller subset of the interface (“hot-spots”) [31] for review, might be responsible for the high affinity protein/protein interaction. It appears that in the case of uPAR/integrin interaction, serine 245 of a small α-helix is located in a surface exposed cavity that provides a crucial site for molecular interaction (see Fig. 4A). Polar atoms of the protein located within 3Å of MS#479, (Fig. 4B) suggest possible derivatives for improvement of its affinity. Since the lead compounds were identified from a diversity library of compounds selected on the basis of the Lipinski rule for drug-like properties, we expect that it will be possible to further optimize our lead compounds by chemical modifications.

In summary, we have found a potential cancer specific target, uPAR/integrin interaction site, and used it to identify a small molecule that, by disrupting this interaction, might be able to force cancer cells into dormancy. This might provide opportunity to convert residual disease in cancer patients into a chronic but asymptomatic state.

## Materials and Methods

An ethics statement is not required for this work. *Reagents and Antibodies*- BSA and human FN were purchased from Sigma Chemical Co. (St.Louis, MO), Steady Glo lysis buffer from Promega, (Madison, Wisconsin), Aprotonin and trypsin, from ICN Biomedicals, Inc. (Aurora, OH), DMEM, glutamine, antibiotics and Lipofectin from LifeTechnologies, Inc. (Grand Island, NY) and FBS from JRH Biosciences (Lenexa, KS). Anti-P-ERK1/2 (anti-phospho-tyr-204, clone E4) was from Santa Cruz Biotechnology (Santa Cruz, CA), anti-ERK1/2 (clone MK12) from Transduction Laboratories (Lexington, KY), anti-α5β1(HA5) and rabbit anti-α5 antibodies from Chemicon International (Temecula, CA), Alexa Fluor 488 F(ab′)_2_ fragment of rabbit anti-mouse IgG (H+L) and Neutravidin HRP from Molecular Probes, Invitrogen (Carlsbad, CA), monoclonal anti-uPAR domain III (R2) and domain I (R3) antibodies were a gift from Dr. M. Ploug (Finsen Institute, Copenhagen). COFAL-negative embryonated eggs were from Specific Pathogen-Free Avian Supply (North Franklin, CT); protein G-agarose beads were from Roche Molecular Systems Inc., (Branchburg, NJ). FN-depleted serum was prepared on a gelatin-Sepharose4B column as per manufacturer instruction. The compounds for testing were provided by the Translational Chemical Biology Center (TCBC), Mount Sinai School of Medicine.

### Computer Screening of compounds

A diversity library of about 13,000 small molecules selected on the basis of the Lipinski rule for drug-like properties [7] was screened *in-silico* using Autodock (v 3.0.5) for possible binders to uPAR, targeting the region comprising residues 240–248. The input describing the protein was prepared with the program ADT (Autodock Tools) that add charges and non-bonded parameters to the protein structure file. The protein was further oriented to minimize the enclosing rectangle using an in-house program Simulaid (developed by M. Mezei). The screening was driven by a script that runs the docking of several ligands on a cluster of CPUs in parallel, allowing the full screening to be completed in a couple of weeks. The docked poses were sorted and the top-scoring molecules were tested experimentally by using biochemical and cell-based assays as described above.

### Test of top-scoring compounds for ERK inhibition using ELK-luciferase or Western blot for phospho-ERK

T-HEp3 cells [32] were transiently co-transfected with pFA-Elk1-fusion plasmid and pD700 -luciferase plasmid as previously described [33]. These plasmids report through Luciferase activity level, on the state of ERK- activation. After 24 hrs cells were serum-starved for 5 hrs, and treated with 5 or 50 µM of PD98059 or 5 µM of the test compound dissolved in DMSO and diluted in DMEM. Following 16 hr incubation, the cells were lysed directly in Steady glo-luciferase lysis buffer (1∶1) from Promega and Luciferase activity measured using Tecan Safire2™ and Magellan 6.0 software. To analyze the effect on P-ERK, T-HEp3 cells were serum starved and treated with 20 µM PD98059 or compounds for 20 min, lysed in RIPA buffer (1% Triton X-100, 0.1%SDS, 10 mM Tris pH 8.0, 140 mM NaCl and protease inhibitors) for 30 min on ice, centrifuged and the supernatants (20 µg protein) were analyzed by Western blotting using anti-P-ERK and anti-ERK antibodies.

### Disruption of FN- fibrils

T-HEp3 cells were suspended in 5% FN-depleted FBS/ DMEM without or with 10 µM compound MS#479, MS#305, MS#304, MS#128, or anti-uPAR antibody (R2, 20 µg/ml), incubated for 15 min at room temperature, seeded in chambered slides and incubated at 37°C. After 1 hr, medium with 10 µg/ml of human FN was added and the cells were fixed and stained for FN after additional 16 hrs of incubation. The images were observed in fluorescent Nikon Eclipse E600 microscope and photographed with SPOT-RT™ camera, Spot Diagnostic Instruments (Sterling Height, MI).

### Inhibition of β1 integrin activity examined by FACS analysis

T-HEp3 cells incubated with compound MS#304, MS#305 and MS#479 (5 and 50 µM) or MnCl_2_ (0.5 mM) for 20 mins, were detached, resuspended in DMEM and aprotinin (20 µg/ml) at 5×10^5^ cells/100 µl and incubated with antibody (5 µg/ml) to active β1-conformation (HUTS-4) or isotype matched (IgG2b) IgG at 37°C for additional 15 min in the presence of compounds, followed by rabbit anti-mouse Alexa 488-coupled IgG (1∶400) at 4°C for 25 min, suspended in FACS buffer (1% BSA in PBS with 10 µg aprotinin) and analyzed in FACS Canto (Becton Dickinson, CA) using FACSDiva software. The numbers show mean fluorescence intensity of HUTS-4 as percent of untreated control.

### Adhesion Assay

T-HEp3 cells were tested for adhesion as previously described [5]. Briefly, cells were mixed with 15 µg/ml anti-uPAR antibody (R2), (positive control) or the compounds MS#479, MS#305 at 5, 10 and 20 µM and compound MS#410 at 20 µM before inoculation on wells pre-coated with 0.4 µg/ml of fibronectin. Following 15 min incubation at 37°C, the cells were processed as previously described [5]. The graph represents mean (±S.D.) of four determinations for each sample.

### Disruption of uPAR/α5β1-integrin complex

Surface biotinylated T-HEp3 cell lysates were prepared as described [5] and 1 mg protein per sample was IP-ed with 5 µg of anti-α5β1(HA5), anti-uPAR (R3) antibodies, or isotype-matched IgG bound to G-agarose beads. Compounds MS#479 and MS#410 at 2.5 or 5 µM were added for the last 15 min of IP. The bead-bound proteins were analyzed as previously described [5]. The upper panel of the blot was stripped and re-probed with rabbit anti-α5 antibody. The bands were scanned and quantified using Image J and the uPAR associated with α5-integrin represented as percent of the diluent treated control.

### Effect of compounds on tumor growth and metastasis

T-HEp3 cells were stably transfected with plasmids reporting for active ERK as described for Fig. 1B except that in some experiments the reporter was GFP. Cells (4×10^5^ /30 µl) in PBS with 0.05% DMSO, 10 µM of MS#410 (control), or MS#479 or MS#305 were inoculated into 8 mm Teflon rings placed on CAMs of 10 day old chick embryos, 4 CAMs per group and treated daily with 30 µl PBS/DMSO or PBS/compounds for 5 consecutive days. Tumors were weighed, and either minced and lysed for Luciferase activity measurements or, when generated by T-HEp3-ELK-GFP- cells, dissociated into single cell suspensions and total tumor cells and GFP-expressing cells were counted using Nikon TS100 fluorescent microscope. For the effect of compounds on spontaneous metastasis, the embryo lungs from the above experiment were dissected and the DNA was extracted and used to amplify a 220 bp fragment of human *alu* sequence as we previously described [21].

## Supporting Information

Figure S1 Structure of compounds that docked on uPAR but did not inhibit ERK nor disrupt fibrils. A library of compounds was docked on uPAR (as described in Material and Methods) and those that docked were examined for their ability to inhibit ERK using ERK-luciferase HEp3 tester cells (see Material and Methods), and for their ability to disrupt cell surface fibronectin fibrils (as a measure of α5β1-integrin inactivation, see Material and Methods for details). The structures of compounds without inhibitory activities are depicted here. Compounds that inhibited ERK were tested further.
(0.09 MB TIF)
