# Supplementary figures and images for: Computer Aided Identification of Small Molecules Disrupting uPAR/α5β1- Integrin Interaction: A New Paradigm for Metastasis Prevention
Source: PLoS One. 2009 Feb 26;4(2):e4617. doi: 10.1371/journal.pone.0004617 (PMC2643475; doi:10.1371/journal.pone.0004617)

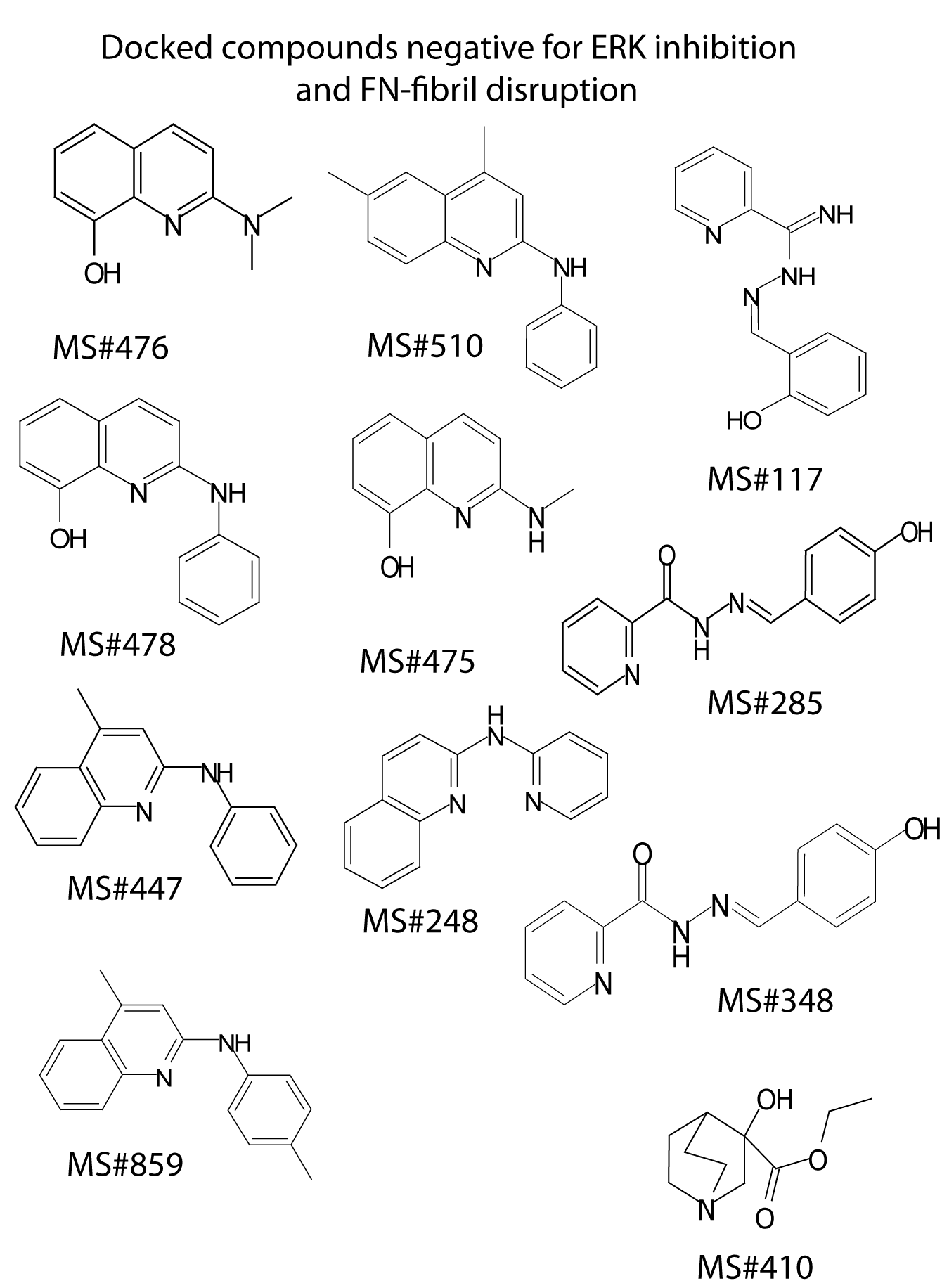

Supplement: Figure S1 — Structure of compounds that docked on uPAR but did not inhibit ERK nor disrupt fibrils. A library of compounds was docked on uPAR (as described in Material and Methods) and those that docked were examined for their ability to inhibit ERK using ERK-luciferase HEp3 tester cells (see Material and Methods), and for their ability to disrupt cell surface fibronectin fibrils (as a measure of α5β1-integrin inactivation, see Material and Methods for details). The structures of compounds without inhibitory activities are depicted here. Compounds that inhibited ERK were tested further. (0.09 MB TIF) [file pone.0004617.s001.tif]
